# Minimally Invasive Surgery for Intracerebral and Intraventricular Hemorrhage

**DOI:** 10.3389/fneur.2022.755501

**Published:** 2022-02-22

**Authors:** Zelong Zheng, Qi Wang, Shujie Sun, Jinbiao Luo

**Affiliations:** ^1^The Department of Neurosurgery, Guangzhou First People's Hospital, School of Medicine, South China University of Technology, Guangzhou, China; ^2^Institute of Eco-Environmental and Soil Science, Guangdong Academy of Sciences, Guangzhou, China; ^3^Shanghai Clinical Research Centre of Chinese Academy of Sciences, Shanghai, China

**Keywords:** intracerebral hemorrhage, intraventricular hemorrhage, minimally invasive surgery, mechanical-based approach, pharmacological catheter-based approach

## Abstract

Spontaneous intracerebral hemorrhage (ICH), especially related to intraventricular hemorrhage (IVH), is the most devastating type of stroke and is associated with high mortality and morbidity. Optimal management of ICH remains one of the most controversial areas of neurosurgery and no effective treatment exists for ICH. Studies comparing conventional surgical interventions with optimal medical management failed to show significant benefit. Recent exploration of minimally invasive surgery for ICH and IVH including catheter- and mechanical-based approaches has shown great promise. Early phase clinical trials have confirmed the safety and preliminary treatment effect of minimally invasive surgery for ICH and IVH. Pending efficacy data from phase III trials dealing with diverse minimally invasive techniques are likely to shape the treatment of ICH.

## Introduction

Spontaneous intracerebral hemorrhage (ICH) is the most serious public health issue and impinges ~2 million people in the world each year (1–3). The incidence of ICH has been increasing because of an aging population and increased use of anticoagulation and antiplatelet agents for thromboembolic diseases. Although ICH accounts for 6.5–19.6% of strokes, it is related to the high rate of mortality and morbidity; the 30-day mortality rate is in the range of 35–52% and only 20% of survivors live independently (4). When ICH involves intraventricular hemorrhage (IVH), the outcome is even worse, with an estimated mortality rate of between 50 and 80% (3). Since most cases occur in working adults in a large number of people in low- and middle-income countries, ICH has a huge social and economic impact due to the loss of productive life years (5).

Risk factors for ICH include hypertension, anticoagulation, and amyloid angiopathy. Most patients (50–70%) with ICH suffer from hypertension (6). Hypertensive hemorrhage is inclined to occur in the external capsule (42%), pons (16%), thalamus (15%), cerebellum (12%), and white matter (10%) (7). Evidence from studies indicates that the incidence of ICH is reduced due to improved control of hypertension (8, 9). Anticoagulation-related ICH is more severe and associated with more extensive hemorrhage and a higher mortality rate in comparison with non-anticoagulation-related ICH and has risen in incidence over the past decade (10, 11). Cerebral amyloid angiopathy (CAA) is associated with 12–15% lobar ICH, particularly in the elderly (12, 13). CAA-related ICH usually occurs in people aged > 60 years. Compared with other causes of ICH, CAA-related ICH has a lower mortality rate, but an increased rate of recurrence (14).

Unfortunately, there has been no beneficial medical treatment for ICH and surgery lacks definitive evidence and is still in debate. Theoretically, hematoma evacuation could remove the neurotoxic blood products, reduce the mass effect and intracerebral pressure (ICP), and control cerebral perfusion pressure, helping to prevent brain edema and secondary brain injury. Open surgery, however, may make damage to the normal brain tissue and affect functional outcomes, especially for patients with deep hemorrhage. The International Surgical Trial in ICH (STICH) and STICH II were the two largest prospective randomized clinical studies to evaluate the efficacy of open surgery for ICH. These clinical trials did not find a significant benefit in functional outcomes between patients who underwent surgery and patients who were medically treated (15, 16). The failure of traditional surgical treatment to improve the prognosis of patients with supratentorial ICH is because, in many patients, the damage of surrounding brain tissues caused by surgical methods neutralizes the benefits of hematoma clearance. This makes minimally invasive surgery (MIS) the most promising surgical strategy for patients with ICH. Because of the shorter operation time, the possibility of bedside treatment, and less damage to the brain tissue, minimally invasive treatment is more attractive than conventional craniotomy in the treatment of ICH (17).

In this review, we discuss different minimally invasive techniques for ICH and IVH, emphasizing clinical trials (Table 1) for this condition.

**Table 1:** Randomized controlled trials of MIS for ICH and IVH.

**References**	**Treatment**		**Included patients** **(MG/OG)**	Age^*^ (years) **(MG/OG)**	**Hematoma** **location**	Volume^*^(ml) **(MG/OG)**	**Onset to** **surgery (hours)**	**Outcomes**	**Follow-up** **(months)**
	**MG**	**OG**							
Auer et al. (18)	ES	CMT	100 (50/50)	30–80	Subcortical, putaminal, or thalamic	≥10	Within 48	➀➁➂	6
Naff et al. (19)	EVD with urokinase	EVD with saline	12 (7/5)	49.6/55.2	IVH with or without supratentorial ICH	ICH: 5.3/13.2 IVH: 72.8/41.54	NA	➁➂➃➄	1
Teernstra et al. (20)	SA with urokinase	CMT	71 (36/35)	67/69	Supratentorial	66/52	Within 72	➀➁	6
Zhang et al. (21)	ES with EVD	EVD with urokinase	42 (20/22)	31-75	IVH with or without Supratentorial ICH	ICH < 30	Within 48	➀➁	2
Kim and Kim (22)	SA	CMT	387 (204/183)	64.3/67.1	Basal ganglia and thalamus	24.3/21.0	NA	➀➁➂	6
Wang et al. (23)	CP with urokinase	CMT	377 (195/182)	56.6/56.9	Basal ganglion	33.9/31.3	4–72	➀➁➂	3
Chen et al. (24)	ES with EVD	EVD	48 (24/24)	65.54/62.17	IVH with thalamic hemorrhage	ICH:10.5/11.5	NA	➀➁➄	3
Sun et al. (25)	CP with urokinase	CC with small bone flap	304 (159/145)	56.9/55.2	Basal ganglion	52.3/51.7	Within 72	➀➁➂	3
Naff et al. (26)	EVD with rt-PA	EVD with placebo	48 (26/22)	54.1/56.6	IVH with or without supratentorial ICH	ICH: 7.2/7.9 IVH:54.8/50.1	≤ 12	➁➂➃	1
Zhou et al. (27)	CP with urokinase	CC with large bone flap	168 (90/78)	57.6/59.2	Basal ganglion or brain lobe	30–100 ml	6–24	➀➁➂	12
Hanley et al. (28)	SA with rt-PA	CMT	96 (54/42)	60.7/61.1	Lobar or deep	48.2/43.1	NA	➀➁➂	12
Vespa et al. (29)	ES	CMT	24	59/62	Supratentorial	36.4/41.4	Within 48	➀➁➂	12
Hanley et al. (30)	EVD with rt-PA	EVD with saline	500 (249/251)	59/59	IVH with or without supratentorial ICH	ICH:8.3/7.2 IVH:21.2/22.4	≤ 12	➀➁➂➃➄	6
Hanley et al. (31)	SA with rt-PA	CMT	506 (255/251)	62/62	Basal ganglia or lobar region	42.7/41.5	NA	➀➁➂	12

*CC, conventional craniotomy; CMT, conservative medical treatment; CP, craniopuncture; ES, endoscopic surgery; EVD, external ventricular drainage; ICH, intracerebral hemorrhage; IVH, intraventricular hemorrhage; MG, minimally invasive surgery group; NA, not available; OG, other treatment options group; SA, stereotactic aspiration. ➀ good functional outcome; ➁ death; ➂ rehemorrhage; ➃ ventriculitis; ➄ ventriculoperitoneal shunt*.

* *Age and volume are usually expressed as mean or median. If there was no such information in literatures, a range would be given*.

## Minimally Invasive Surgery for ICH

The use of MIS for ICH evacuation started in the 1960s (32). The endoscope was used to evacuate the clot after ICH (33). Since then, several improvements have been made to the method to improve the efficiency of hematoma removal (34). In the late 1980s, mechanically-assisted thrombolysis by using ultrasound techniques was explored and favorable outcomes were described (35). MIS continues to evolve with the advancement of technology. Generally, MIS approaches can be divided into two categories: primarily pharmacological catheter based and primarily mechanically based. The pharmacological catheter-based approach involves the placement of a drainage catheter under the guidance of images and gentle aspiration for hematoma, followed by the infusion of the catheter with a thrombolytic agent to prevent clogging, facilitating passive drainage of the hematoma over several days. This requires intermittent imaging to confirm the catheter position and to monitor the progress of hematoma drainage. Mechanical methods for hemorrhage evacuation involve the surgical removal of the hematoma in a single procedure without leaving a drainage catheter and by using a thrombolytic agent. The evacuation of the hematoma is performed with either an endoscope or other devices. These two approaches have their own advantages and disadvantages, which may or may not contribute to functional outcomes in patients with ICH.

### Pharmacological Catheter-Based MIS for ICH

**Figure 1:**
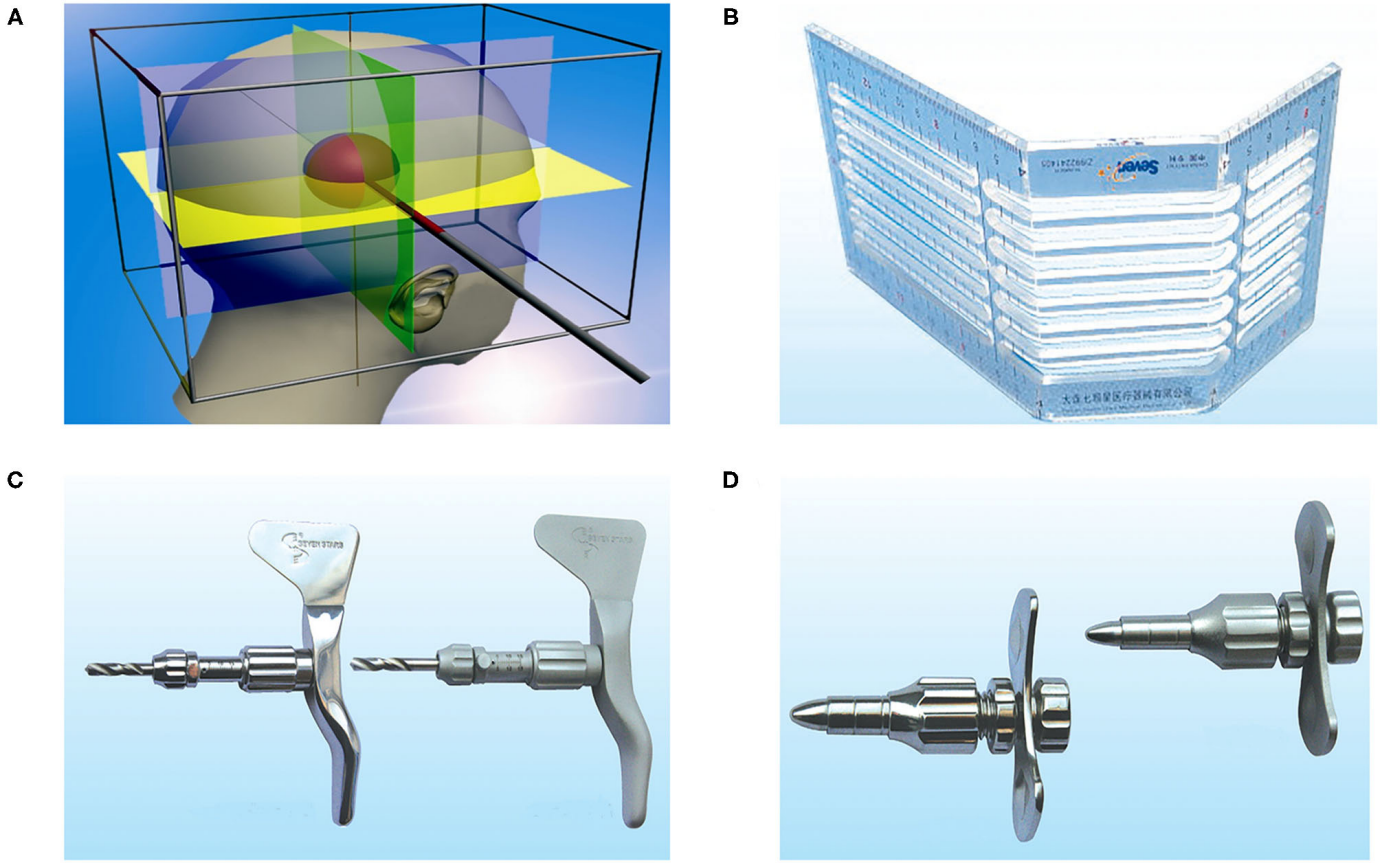
The principle and devices of cubic oriented stereotactic aspiration technology. **(A)** The principle of cubic oriented stereotactic aspiration: according to the principle of solid geometry, the position of any point in space can be determined by the three-dimensional directional coordinate system. Combining this principle with cranial anatomy, the “quasi-circular” head is “framed” in cube space. According to the principle that three mutually perpendicular planes intersect to form a three line and a point in a cube, a horizontal plane, a coronal plane, and a sagittal plane passing through the point can be made, respectively, to form a three-dimensional directional coordinate system with this point as the origin. According to the parameters of CT scanning, the vertical projection lines and planes in the forehead, temporal part, and top and occipital parts were determined. Then, the center of the hematoma was determined according to the intersection of the three planes. The straight line formed by the intersection of any two planes in the three mutually perpendicular planes can be used as the puncture path and the position of the other plane can determine the puncture depth. This allows accurate access to the center of the hematoma. The stereotactic ruler **(B)**, skull drill **(C)**, and skull keyhole tool **(D)** are used in cubic oriented stereotactic aspiration.

**Figure 2:**
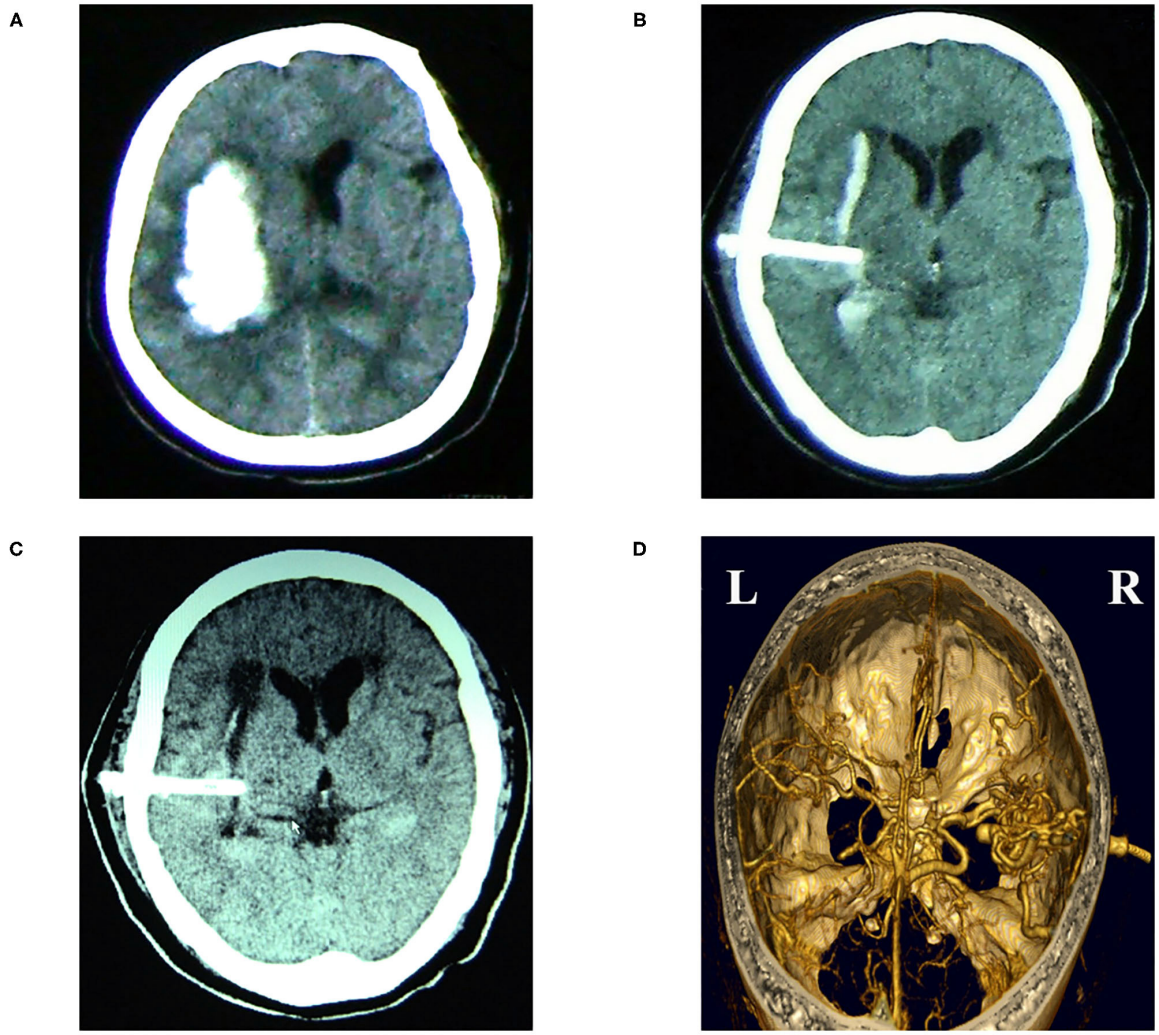
Images from a 65-year-old patient with the hypertensive right temporal hemorrhage who received cubic oriented stereotactic aspiration. **(A)** Preoperative CT image of the head. **(B)** A post-operative CT image of the head was carried out immediately, which showed that the soft drainage tube was inserted into the hematoma and the volume of the hematoma was reduced after aspiration. **(C)** CT image at 4 days after operation displayed that the hematoma was almost completely evacuated. **(D)** The post-operative CT angiography image of the head showed that the soft tube did not lead to the damage of vessels.

Matsumoto and Hondo reported that they performed CT-guided stereotactic evacuation of hypertensive intracerebral hematoma in 51 patients. First, they inserted one silicon tube into the center of the hematoma and then, the hematoma was aspired with a syringe, followed by the administration of urokinase until the hematoma was completely evacuated. Of 51 patients, 38 patients had functional outcomes (36). This method was also used in the posterior fossa hematoma. In one study, the aspiration and administration of urokinase were performed in 11 patients (9 patients with cerebellar hematoma and 2 patients with pontine hematoma) and 7 patients had functional outcomes (37). Thereafter, the recombinant tissue-type plasminogen activator (rt-PA) was used for clot thrombolysis and it was well tolerated and more effective in evacuating hematoma (26, 38). In addition, sonothrombolysis, in combination with pharmacological thrombolysis, has been used for clot evacuation. Newell et al. reported that rt-PA and 24 h of continuous ultrasound were delivered to the clot in 33 patients with ICH and they found that the combination of sonothrombolysis and rt-PA was more effective than rt-PA alone in hemorrhage lysis (39). Recently, Sun and Liu developed a new catheter-based MIS technology called cubic oriented stereotactic aspiration (40) and invented associated devices. This technology combines brain anatomy with solid geometry (Figure 1). Luo et al. confirmed the safety and efficacy of the technology (41) (Figure 2). At present, this technology has been used for supratentorial and infratentorial ICH in China (42–44).

These preliminary studies have shown that pharmacological catheter-based MIS can effectively remove hematomas. However, there is no unified standard for the selection of patients, the timing of MIS, and the proportion of hematoma evacuated. More importantly, the clinical benefit is still unclear. Therefore, it is essential to conduct randomized controlled trials (RCTs) to evaluate the effect of the pharmacological approach of MIS.

#### YL-1 Craniopuncture

Wang et al. randomized 377 patients with 25–40 ml basal ganglion hematoma in a multicenter controlled study into conservative medical treatment or the stereotactic craniopuncture and aspiration with YL-1 puncture needle followed by infusion of urokinase. Although there were significantly more complications in the craniopuncture group, the case fatality in these two groups was not significantly different and MIS led to a significant reduction of dependence at the end of the third month, compared with the conservative treatment (40.88 vs. 63.03%) (23). Moreover, other two RCTs were performed to evaluate and compare the effect of YL-1 craniopuncture plus urokinase with craniotomy with a small bone flap or conventional surgery in patients with ICH. Compared with craniotomy with a small bone flap and conventional surgery, the MIS had fewer complications and had a trend to improve long-term outcomes (25, 27). These studies suggest that minimally invasive craniosurgery combined urokinase is safe and might improve independent survival in patients with ICH. Nonetheless, the YL-1 needle is sharp and rigid, which may induce damage to brain tissues and rebleeding. Therefore, large-scale RCTs are needed to evaluate the safety and efficacy of YL-1 craniopuncture with thrombolytic agents.

#### Stereotactic Treatment of Intracerebral Hematoma by Means of a Plasminogen Activator (SICHPA)

The SICHPA trial was a multicenter RCT to investigate the efficacy of stereotactic treatment of ICH with the use of a plasminogen activator. This trial enrolled 71 patients with ICH volume ≥ 10 ml and randomly assigned them to surgery with catheter placement and urokinase infusion or medical treatment. The SICHPA significantly reduced the ICH volume. However, the rebleeding rate and mortality at 180 days were similar in these two groups (20). This study suggests that stereotactic aspiration is safe and effective for reducing the ICH volume. Nevertheless, the number of patients enrolled in this trial is small and, thus, the results should be interpreted carefully.

#### Minimally Invasive Surgery Plus Recombinant Tissue Plasminogen Activator in Intracerebral Hemorrhage Evacuation (MISTIE)

The MISTIE was an international, randomized, open-label, phase II study that enrolled 96 patients with ICH from 26 hospitals and randomized them into medical care or MIS with alteplase. According to the MISTIE protocol, a rigid cannula was inserted into the hematoma under the image guidance and then one soft drainage cannula was placed. The administration of rt-PA in a dose of 0.3 or 1.0 mg was carried out every 8 h until 9 doses were given or until the volume of remaining hematoma was ≤ 15 ml or until a clinically significant rebleeding occurred. MIS plus alteplase decreased the number of patients with the modified Rankin Scale (mRS) ≥ 3 in 1-year follow-up and significantly decreased the perihematoma edema volume. In addition, 30-day mortality, infection, and symptomatic bleed were not significantly different between MIS plus alteplase and the conservative groups. Moreover, the volume of hematoma remaining was statistically significantly correlated with functional outcomes when other variables were controlled. These results suggest that MIS plus alteplase is safe to effectively evacuate hematoma and may improve functional outcomes through reducing clot volume (28). However, the sample size is relatively small and the objective of this study is to detect the threshold of safety not efficacy. In addition, the wide range of benefits estimated in intention-to-treat analyses makes it difficult to draw conclusions about benefits.

To test the conclusion of the MISTIE II and ensure its generalizability, the MISTIE III was performed in 78 centers. Appropriate patients were ≥ 18 years with supratentorial hemorrhage more than 30 ml plus the Glasgow Coma Scale (GCS) ≤ 14 or the National Institutes of Health Stroke Scale (NIHSS) score ≥ 6 and the hematoma remained stable (growth < 5 ml) for at least 6 h after diagnostic CT. A total of 506 patients were enrolled in this study. The process for cannula placement and rt-PA injection was the same as described in the MISTIE II, except that the dose of rt-PA was different and limited to 1.0 mg. The mean reduction in hematoma size was 69% in the MISTIE group vs. 3% in the standard medical care group. Nonetheless, only 58% of patients met the preset goal that the hematoma was <15 ml after surgical treatment and the functional outcome between these two groups did not significantly different. In addition, the safety events rates were not significantly different between the two groups (31). The main conclusion of this clinical study is that the MISTIE is safe and effective for ICH evacuation, but it cannot improve the prognoses of patients. Therefore, it cannot be recommended as an intervention to improve the functional outcome of all the patients with ICH. However, the MISTIE may improve functional outcomes when the protocol-defined surgical aim can be achieved (residual hematoma smaller than 15 ml) (31). In an exploratory analysis, reduction of hematoma volume to ≤ 15 ml associated with a good functional outcome (mRS 0–3) and reducing the hematoma volume by 70% or more increased the chance of achieving a good outcome, i.e., if the reduction exceeded the 15 ml threshold, the probability of good results would increase by 10% for every additional 1 ml of hematoma removed. The failure to achieve ≤ 15 ml goal evacuation was significantly associated with initial hematoma volume, history of hypertension, irregular-shaped hematoma, number of alteplase doses given, surgical protocol deviations, and catheter manipulation problems. Additionally, greater surgeon/site experience was associated with avoiding poor hematoma clearance. Taken together, the results indicate that surgical performance may determine the outcomes of patients with ICH. Enhanced surgical performance may improve the prognosis of patients.

### Mechanically-Based Approaches for ICH

#### Endoport-Mediated Evacuation

The endoport system for clearing ICH originates from the computer-aided stereotactic method for resection of deep and central intracranial tumors. The system aims to effectively remove the hematoma and minimize damage to adjacent tissues. The endoport-mediated evacuation involves a small craniotomy with dura opening, stereotactic placement of an endoport to the hematoma (typically along the longest axis of the hematoma), and removal of hematoma with traditional surgical tools or special devices. Compared with the pharmacological catheter-based approach, this technique allows direct access to the hematoma cavity with irrigation, suction, and cautery instruments and clears more hematoma at once. It, however, needs craniotomy and may increase brain damage along the endoport path.

**Figure 3:**
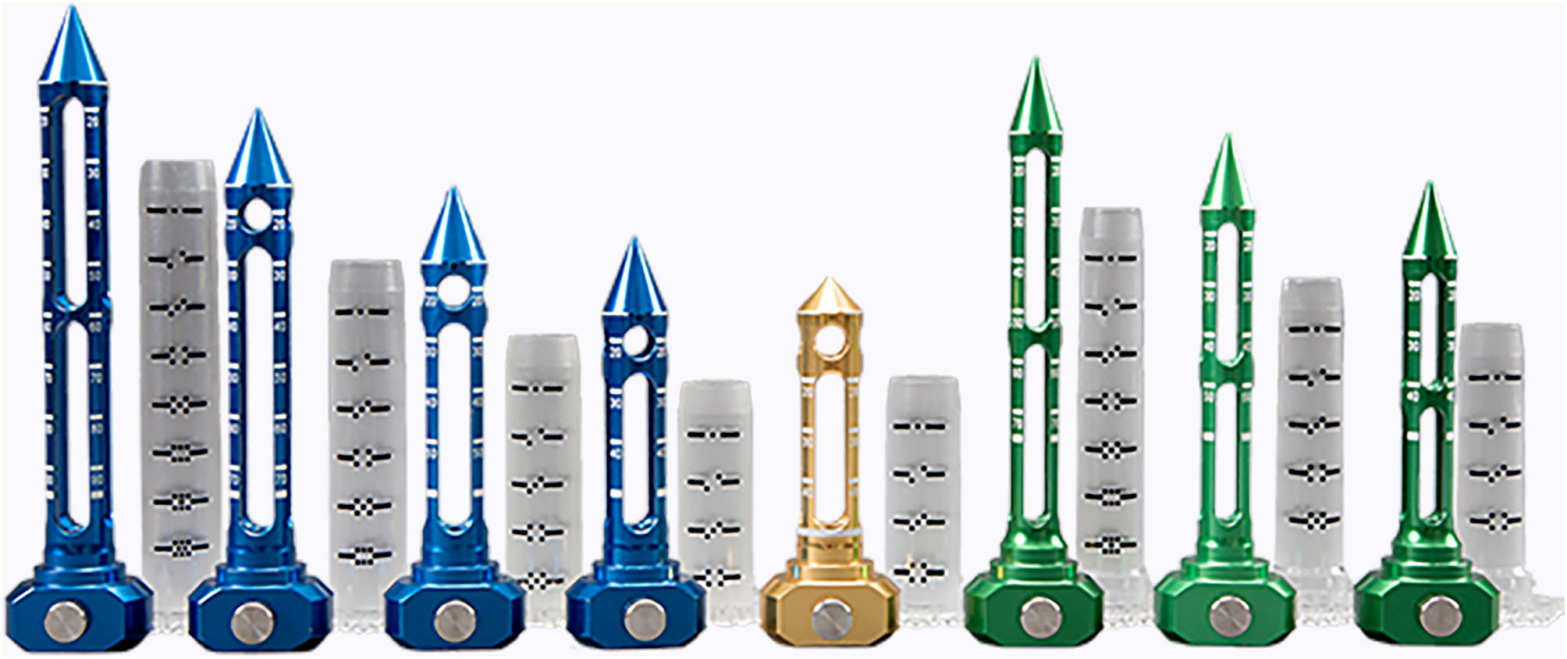
The NICO BrainPath System consists of a 13.5 mm diameter sheath with an internal dilator. The sheath and dilator have different lengths.

Currently, the most commonly used device is the NICO endoport system (Indianapolis, Indiana, USA). The NICO endoport system, consisting of the Myriad handpiece and the BrainPath sheath (Figure 3), is cleared by the United States Food and Drug Administration for the removal of tissue and visualization of the surgical field during neurosurgery (45). Ding et al. presented a case report about the use of NICO endoport system to evacuate a large basal ganglia hematoma (46). And then, they performed a retrospective study of 11 patients with ICH (9 with supratentorial hemorrhage and 2 with infratentorial hemorrhage and the volume of ICH from 8 to 168 ml) and used the same technique to evacuate the hematoma. They found that the ICH volume was reduced by 87% and 36% of patients were functionally independent (mRS 0–2) at 90 days (47).

##### Minimally Invasive Subcortical Parafascicular Access for Clot Evacuation (MISPACE)

A multicenter study to evaluate the safety and feasibility of the MISPACE by using the NICO endoport system was conducted in 11 centers in the United States and Canada. A total of 39 patients with ICH (ICH volume from 27 to 65 ml) were enrolled. The result of this study showed that hematoma volume was reduced by more than 90% in 72% of patients and 52% of patients had good functional outcomes with the mRS ≤ 2 at 90 days (48). The authors concluded that this method was safe and feasible to remove the clot. However, the sample size of this study is small and it is a retrospective, uncontrolled study. RCTs are still needed to assess the safety and effectiveness of the MISPACE.

##### Early MiNimally-Invasive Removal of Intracerebral Hemorrhage (ENRICH)

The ENRICH is a multicenter randomized controlled study comparing early (<24 h) surgical hematoma evacuation by using minimally invasive parafascicular surgery (MIPS) with the NICO endoport system with standard medical management in the treatment of acute spontaneous supratentorial ICH. This study is based on the solid foundation supplied by the results from the multicenter study for the MISPACE and other clinical studies by using the NICO system. The ENRICH is designed to enroll 300 patients with ICH and was started in 2016. It is still in progress.

#### Endoscope Evacuation

The ICH evacuation by an endoscope with the use of a single burr hole was first reported by Auer et al. (49). Thereafter, the same group performed a single-center randomized controlled study that compared endoscopic evacuation vs. medical treatment in patients with ICH. A total of 100 patients with supratentorial hematoma more than 10 ml and neurological or consciousness impairment were enrolled and assigned randomly. The clearance rate of hematoma in most patients was 50–70%. The mortality rate in the surgical group was significantly lower than the medical treatment group 6 months after hemorrhage (42 vs. 70%). The rate of better outcomes was higher in the surgical group than in the medically treated group (40 vs. 25%). However, these results were restricted in patients under 60 years old and with hematoma smaller than 50 ml (18). Since these promising results were obtained by the first generation of endoscopy, it is difficult to transfer the findings to the clinical situation of today. A more recent retrospective analysis of 68 patients with supratentorial ICH treated with endoscope-assisted ICH evacuation showed that rebleeding, morbidity, and mortality rates were low and long functional outcome was favorable when compared with the traditional craniotomy (50). Moreover, another retrospective analysis of 43 patients with putamen hematoma (volume > 31 ml), cerebellar ICH (hematoma > 3 cm in diameter), or thalamic ICH (hematoma volume > 20 ml) and acute hydrocephalus treated with endoscopic surgery and craniotomy procedure demonstrated higher evacuation rate and the higher GCS score at day 7 in the group of endoscopic surgery (51). However, the safety and efficacy of endoscopic surgery for ICH evacuation should be evaluated in RCTs.

##### Intraoperative Stereotactic CT-Guided Endoscopic Surgery (ICES) Trial

The ICES trial was a randomized arm of the MISTIE trial. In this study, 24 patients with hematoma volume more than 20 ml from different hospitals were randomized 3:1 to endoscopic surgery or standard medical management. The result showed that endoscopic surgery led to a 71.2% reduction of ICH with an associated 12% increase in good functional outcomes defined as the mRS score from 0 to 3 at 1 year (29). Although the number of patients involved in this preliminary study was small, the result of the study suggests that the endoscopic technique can be performed by different surgeons in a different location and can produce valuable results worthy of further evaluation in the phase III study.

##### Apollo System and the Minimally Invasive Endoscopic Surgical Treatment With Apollo in Patients With Brain Hemorrhage (INVEST)

**Figure 4:**
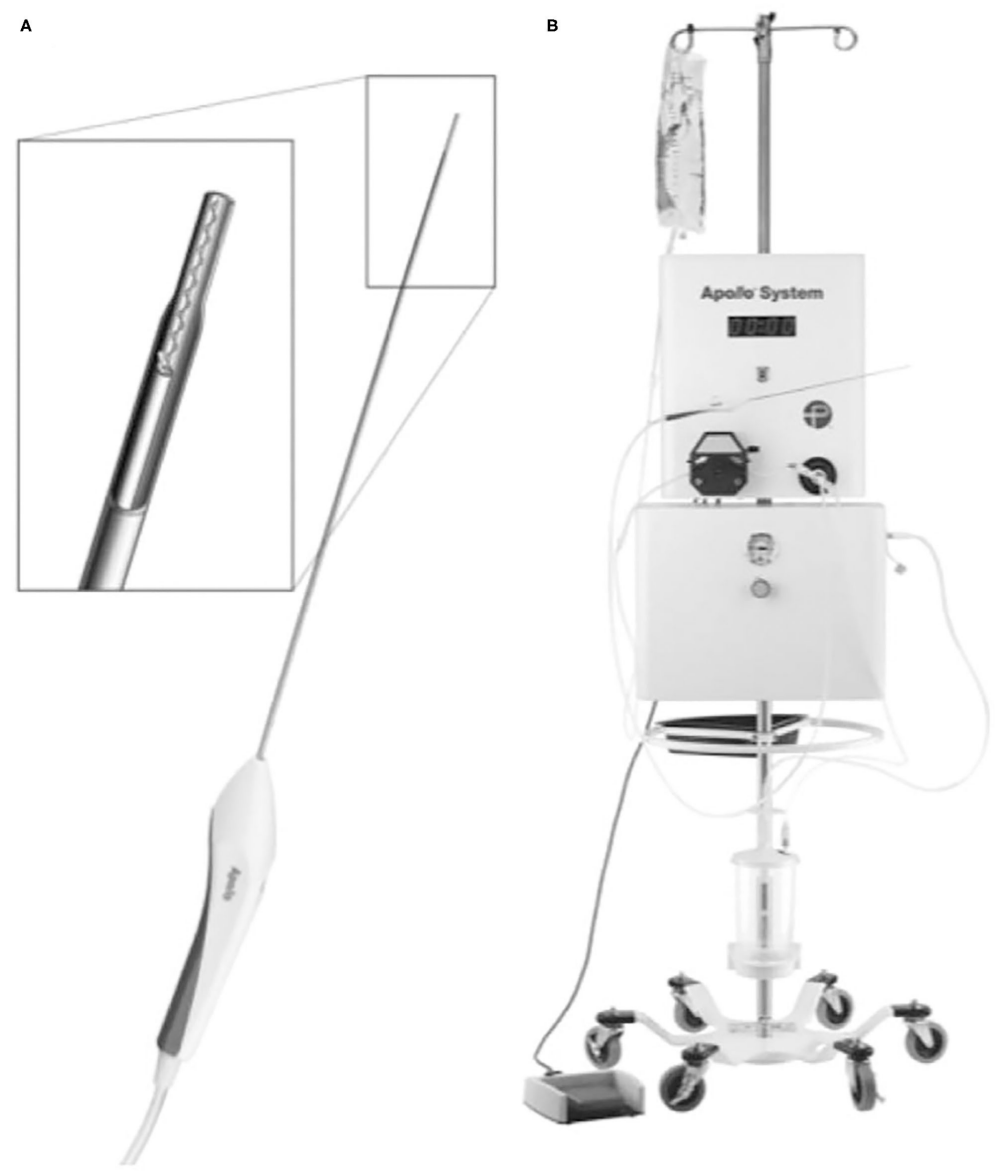
The Apollo System consists of a suction-irrigation system that can be connected to the Apollo wand through a soft tube. The wand **(A)** can be placed through the working channel of a neuroendoscope. The wand is equipped with an internal stirrer line that vibrates at ultrasonic frequency and impregnates the clot material to keep the system unobstructed during suction. The wand is connected to a stand-alone suction-irrigation system **(B)** [from Fiorella et al. (45) with permission].

There are some promising tools used for endoscopic ICH evacuation and the Apollo System (Penumbra Incorporation, Alameda, California, USA) is one of them. The Apollo System is a nonclogging aspiration–irrigation system designed to fit down the working channel of a three-port neuroendoscope (Figure 4). The aspiration–irrigation system can be connected with the Apollo wand through a flexible tube. The wand is equipped with an internal stirrer line that vibrates at ultrasonic frequency and impregnates the clot material to keep the system unobstructed during the suction process. The aspiration–irrigation system provides the ability of suction and continuous saline flushing and transmits vibration energy to the internal agitator elements in the wand. The advantage of the Apollo device in increasing the emptying of the endoscope is that it allows more directional suction and can extend beyond the end of the endoscope in a controlled way. Spiotta et al. performed a retrospective analysis of 29 patients with ICH who underwent ICH evacuation with the Apollo system at 4 centers. The mean ICH volume decreased from 45.4 ± 30.8 to 21.8 ± 23.6 ml after evacuation (52). Fiorella et al. presented case reports of 3 patients with supratentorial ICH who underwent hematoma evacuation by using the Apollo System with combined neuronavigation, neuroendoscopy, and cone-beam CT. The volume of hematoma was reduced from 93.4 to 15.6 ml and 11.2 to 0.9 ml after evacuation. Moreover, no complications occurred for the procedure (53). More clinical data are needed to determine the extent of hematoma removal to improve clinical symptoms and whether it can achieve more complete blood product emptying, thereby offsetting the disadvantages of large channels.

The INVEST trial is a multicenter single-arm phase II trial, which is evaluating the safety and efficacy of the minimally invasive hemorrhage evacuation with Apollo device compared with medical management. The study has initiated enrollment in 2016.

#### Other Mechanically-Based MIS Approach

Kim et al. performed a prospective study, which enrolled 387 patients with ICH volume ≤ 30 ml limited to the basal ganglia and thalamus and randomized them into either MIS by using an Archimedes aspirator or to medical management. If the remaining hematoma exceeded 10 ml after surgical evacuation, the instillation of urokinase in the hematoma cavity was considered. In the MIS group, patients had better functional outcomes with higher mean of the Barthel Index scores (90.9 vs. 62.4) and the lower mRS scores (1.2 vs. 3.0) at 180 days. Meanwhile, the mortality was not significantly different between the MIS and medical treatment groups (22).

## Minimally Invasive Surgery for IVH

Usually, IVH is a complication of spontaneous ICH. However, it sometimes occurs without an identifiable cause. The extension of ICH into the ventricular is an independent risk factor for poor prognosis (54). In addition, the volume of IVH is related to the clinical outcomes (54, 55).

The application of MIS in the treatment of IVH is based on the hypothesis that the reduction of hematoma volume and the restoration of cerebrospinal fluid circulation can reduce secondary brain tissue damage. Like ICH, MIS for IVH includes pharmacological catheter-based and mechanically-based approaches. The pharmacological catheter-based method involves the injection of fibrinolytic drugs (urokinase or rt-PA) through external ventricular drainage (EVD) tube. The mechanically-based approach involves the direct movement of intraventricular hematoma with the endoscopy. These methods have their disadvantages. The disadvantages of the pharmacological catheter-based method include the risk of infection from the repeated injection of a lytic agent through EVD, potential hematoma expansion induced by the lytic agent, a relatively long period for adequately clearing IVH, and risk of failure to reduce the volume of intraventricular hematoma (56). The mechanically-based approach is more invasive and needs craniotomy with a small bone flap.

### Pharmacological Catheter-Based Treatment for IVH

Injection of fibrinolytic drugs through EVD has been proved to be safe and effective in animal studies (57–59). And then, this result has been confirmed in small clinical case series (60–62). Nieuwkamp et al. conducted one systemic review of 343 patients with severe IVH caused by extension from subarachnoid hemorrhage or ICH from 18 observational studies and these patients received conservative treatment, EVD or EVD with fibrinolytic agents. They found that the case fatality rate was significantly lower in patients receiving EVD combined with fibrinolytic agents. The poor outcome rate was also lower for EVD with fibrinolytic agents compared with conservative treatment or EVD. Treatment with EVD combined with fibrinolytic agents may improve the outcome of patients with severe IVH (63). Furthermore, Khan et al. conducted a meta-analysis to investigate the impact of intraventricular fibrinolytic therapy on mortality, functional outcome, ventriculitis, shunt dependence, and rehemorrhage. The meta-analysis enrolled 24 studies and demonstrated the benefit of intraventricular fibrinolytic on mortality and good functional outcome. In addition, intraventricular fibrinolytic decreased the rate of shunt dependence and was not associated with increased rates of ventriculitis or rehemorrhage (64). This meta-analysis concluded that intraventricular fibrinolytic was safe and could be an effective strategy for the treatment of IVH. Nonetheless, those results are from observational or small randomized studies and randomized clinical trials are needed. Then, a randomized, double-blind, controlled, multicenter, pilot study was performed to assess the safety and efficacy of intraventricular thrombolysis. A total of 12 patients with IVH associated with supratentorial ICH of <30 ml were enrolled in this study and randomized to receive intraventricular injections of normal saline solution or urokinase at 12-h intervals. Compared with treatment with EVD alone, intraventricular thrombolysis with urokinase speeded the resolution of intraventricular blood clots and the frequency of adverse events did not differ significantly between the two groups (19). The number of patients enrolled in this study, however, is small and the findings need to be validated in a large study.

#### Clot Lysis: Evaluating Accelerated Resolution of IVH (CLEAR-IVH)

The pilot study led to the CLEAR-IVH trial which was a phase II trial and was to evaluate the safety and efficacy of using multiple injections of low-dose rt-PA to accelerate lysis and evacuation of IVH. In this study, 48 patients with IVH were randomized into treatment with rt-PA and placebo. The administration of rt-PA could accelerate the clearance of IVH clots. Although the symptomatic bleeding rate in patients treated with rt-PA was higher than that in patients receiving placebo, a trend to improve clinical outcomes at 30 days was observed. Moreover, the rate of IVH clot resolution was significantly correlated with early clinical improvement (26). This phase II trial optimized the dose effect of rt-PA and demonstrated a trend toward improved clinical outcome at 30 days in patients treated with rt-PA, promoting a more definitive phase III trial.

The CLEAR III was an investigator-initiated, phase III, randomized, multicenter, double-blind, placebo-controlled study comparing the use of EVD combined with rt-PA with EVD plus saline (placebo) for the treatment of IVH. A total of 500 patients with IVH and obstructive hydrocephalus in 73 centers were enrolled and assigned 1:1 to the alteplase group and the saline group. Alteplase (1 mg) or 0.9% saline was given up to 12 doses, 8 h apart through EVD. The administration of alteplase was stopped when 3rd and 4th ventricles open, intraventricular hemorrhage mass effect relieved, 80% of clot removed, or 12 doses were given. The study showed that intraventricular rt-PA did not affect the primary efficacy outcome, but there were less frequent adverse events and a lower case fatality at 180 days in the alteplase group. In addition, clot removal was significantly related with both the mRS ≤ 3 and case fatality (30). These results suggest that by using alteplase through EVD is safe, but it cannot improve functional outcomes at the mRS 3 cutoff compared with by using saline through EVD. However, the benefits of alteplase may be possible if greater clot removal can be achieved. Unfortunately, only 30% of patients treated with alteplase achieved the goal of 80% blood clot reduction, which may be one of the reasons that alteplase treatment cannot effectively improve functional outcomes.

### Mechanically-Based MIS for IVH Treatment

Several case series and case reports showed the safety and efficacy of endoscopy to treat IVH. Longatti et al. reported 13 patients with spontaneous primary or secondary IVH who underwent endoscopy treatment and they found that endoscopy management was safe and effective to remove the intraventricular blood clot. Compared with other more conventional treatments, endoscopy may improve the clinical outcome of patients with IVH (65). Basaldella et al. performed a comparative retrospective analysis of 96 patients with massive IVH (48 patients treated with endoscopy surgery and 48 patients treated with EVD alone) to assess the efficacy of endoscopy surgery on IVH. The analysis demonstrated that endoscopy treatment combined with EVD reduced the shunting rate by 34% compared with EVD alone. However, endoscopy treatment did not significantly affect the outcome at 1 year as determined by using the mRS (66). In addition, Rienzo et al. presented a retrospective analysis of the two groups of patients with cast IVH who received endoscopy-assisted surgery or EVD and found that endoscope-assisted evacuation reduced ICU staying and cerebrospinal fluid (CSF) clearance times. Moreover, endoscopy seemed to improve neurological outcomes, but without affecting the need for permanent shunt (67). However, these are just experience from small case serials and RCTs are needed to evaluate the safety and efficacy of endoscopy treatment for IVH. Zhang et al. conducted a prospective and randomized study that enrolled 42 patients with IVH and these patients were randomly assigned to the endoscopic group and the EVD group. In the endoscopic group, EVDs were placed after the procedure and if the remaining IVH volumes were > 10 ml, injection of urokinase into the ventricle through EVD was performed. In patients allocated to receive an EVD alone, a lytic solution was also administered through the drain. The authors found that more patients in the endoscopic group got the high Glasgow Outcome Scores at 2 months. The mortality rate was not significantly different between these two groups (21). Chen et al. randomized 48 patients with IVH from thalamic hemorrhage (1:1) into the EVD group and endoscopic surgery followed by the EVD group. In the endoscopic surgery group, a standard suction tube was placed side by side with the endoscope through a hard sheath to evacuate blood from the ventricular system ipsilateral to the thalamus hemorrhage. The endoscopic surgery significantly reduced the length of the intensive care unit (ICU) stay and the rate of the shunt (47.62 vs. 90.48%) compared with EVD alone. Nonetheless, the 30- and 90-day mortality rates and the GCS scores were not significantly different between the EVD and endoscopic surgery groups (24).

Several small randomized and observational studies of endoscopic surgery, EVD alone, or EVD with intraventricular fibrinolysis studies in IVH were assessed in a meta-analysis conducted by Li et al. of 680 patients from 11 studies. This meta-analysis demonstrated that compared with EVD with intraventricular fibrinolysis, endoscopic surgery with EVD significantly reduced mortality and shunt rates and increased hematoma evacuation rate. Therefore, the authors suggested that endoscopic surgery with EVD may be better than EVD with intraventricular fibrinolysis in the management of IVH (68).

In brief, the available evidence suggests that mechanically-based techniques can safely and effectively evacuate IVH. Mechanically-based techniques have the potential to improve functional outcomes in some patients with IVH, but more evidence is needed. Zhu et al. are performing a prospective, randomized, controlled, multicenter clinical trial to compare the prognosis of patients undergoing endoscopic surgery vs. those undergoing EVD for moderate-to-severe IVH. This clinical trial will enroll 956 patients with moderate-to-severe IVH who will be randomly assigned 1:1 to the endoscopic surgery group or the EVD group and recruitment began in 2020 (69). The results from this trial will provide high-quality evidence for mechanically-based MIS treatment for IVH.

From the abovementioned clinical studies concerning the pharmacologically- or mechanically-based MIS for ICH and IVH, it is clear that MIS is safe and effective in removal of hematoma compared with conventional surgery and medical treatment. However, MIS is not suitable for patients with unstable hematoma. Additionally, phases II and III clinical trials do not supply definitive evidence that MIS can improve the prognoses of patients. There are several explanations for this. First, extended clot dissolution time offsets the main goal to minimize secondary injury induced by ICH, which begins only a few hours after bleeding and progresses over time. Second, removing the bleeding foci by dissolving the blood clot without properly managing the suspected blood vessel may lead to further expansion of the hematoma. Third, each MIS method lacks standardization and, thus, it is difficult to teach technology and generalize the results. Last but not least, surgical performance may be different during MIS. Catheter manipulation problems, surgical protocol deviations, and greater surgeon/site experiences influence hematoma evacuation, while the volume of residual hematoma is associated with functional outcomes. Therefore, generalization of optimal performance with hematoma evacuation will require focused surgeon education, emphasizing technical nuances, better demonstration of experience, and a rigorous definition of benchmarks for successful tasks. Future research should focus on the best way to achieve this goal and case selection may need to be customized separately for various technologies. Besides, since ultra-early surgery results in a higher rate of rebleeding, the timing of surgery may be an important part of the effectiveness of MIS and this factor should be included in the trial design.

## New MIS Technologies

### Three-Dimensional Printing Technology

In the recent years, 3D printing technology has become a hot field of biomedical research; it is a rapid prototyping technology based on the digital model file, which is used to print solid objects with complex geometry through layered processing. For ICH, 3D printing technology is based on the original Digital Imaging and Communications in Medicine (DICOM) data of CT. This technology can help to analyze the shape and location of the hematoma of the patient and design surgical approaches that avoid important areas such as the venous sinuses, functional areas, and frontal sinuses. In addition, during hematoma puncture, the puncture angle and puncture position can be fixed by 3D-printed guide holes to reduce tissue damage caused by inaccurate positioning and repeated punctures. Zhang et al. retrospectively analyzed 12 patients with basal ganglia hemorrhage who underwent 3D-printed model-guided endoscopic evacuation for hematoma. They found that the average evacuation rate of hematoma was 97.2% and all the patients had good outcomes evaluated by functional independence measure scores at 6 months, which suggested that 3D-printed model-guided endoscopic evacuation was effective and safe (70). Additionally, Wang et al. applied a 3D-printed navigation mold in puncture drainage for brainstem hemorrhage and found that 3D-printed technology made the puncture drainage more individualized and precise (71).

### Diffusion Tensor Imaging and MIS

Diffusion tensor imaging has been used to assess the integrity of the white matter tract and predict motor functional outcomes in patients with ICH (72, 73). Wu et al. found that the changes of the cortical spinal tract could be observed intuitively through DTI in patients with ICH and MIS can reduce the damage to the cortical spinal tract led by hematoma and even could restore the cortical spinal tract, which was oppressed and displaced by hematoma (74). Moreover, preoperative DTI can help doctors to clarify the positional relationship between hematoma and white matter tracts and, thereafter, to determine the MIS approach and surgical trajectory. Therefore, DTI may be combined with different MIS technologies to optimize MIS approaches and explore the efficacy of MIS.

### Robot for Neurosurgery

The robot for neurosurgery has been used in clinical studies. It establishes 3D coordinates based on CT or MRI. Then, the mapping relationship between the computer and actual images is established through positioning marks and stereotactic surgery is planned and operated virtually. Finally, the auxiliary positioning and navigation by multisensor intelligent mechanical arms are realized. In addition, the robot can display 3D visualization of cerebral vessels based on multimodal image infusion, which could help to accurately locate the hematoma and plan the hematoma puncture trajectory, avoiding vessels. Wang et al. retrospectively analyzed 17 patients with hypertensive ICH who received robot-assisted frameless stereotactic MIS. They found that the average positioning error was 1.28 ± 0.49 mm and the functional outcome of patients was improved at 3 months (75). Xiong et al. conducted a systematic review to assess the safety and efficacy of robotic surgery for ICH and suggested that compared with conventional surgery or conservative treatment, robotic surgery is safer and more effective (76).

## Conclusion

Spontaneous ICH, especially those related to IVH, is still one of the most fatal and disabling diseases, causing a high-cost burden on society. Extensive practice mode, vague practice standard, lack of proven therapy, and persistent adverse results cast a shadow on its management. So far, it has not been clearly shown that neurosurgical interventions can improve the prognosis of patients with ICH. However, new emerging results of MIS for ICH and IVH have been very encouraging: MIS can reduce the secondary injury after ICH and IVH, thus potentially reducing mortality and dependence. During the process of MIS, surgical performance is important, since it is associated with the outcomes of patients. Additionally, new technologies could improve the accuracy and safety of MIS. Currently, trials of several new minimally invasive approaches for ICH and IVH evacuation are being evaluated and more clear evidence is expected in the near future. Moreover, ongoing clinical trials are expected to change clinical practice by optimizing case selection and surgical tasks and integrating these new tools into treatment.

## Author Contributions

ZZ and QW contributed to the literature review and wrote the manuscript. JL contributed to writing and modifying the manuscript. SS read and checked the manuscript. All authors contributed to the article and approved the submitted version.

## Funding

This study was supported by the Guangdong Basic and Applied Basic Research Foundation, China (2019A1515011273), the General Guidance Project of Guangzhou Municipal Health Commission, China (20201A011015), and the Science Foundation of Guangzhou First People's Hospital, China (M2019023).

## Conflict of Interest

The authors declare that the research was conducted in the absence of any commercial or financial relationships that could be construed as a potential conflict of interest.

## Publisher's Note

All claims expressed in this article are solely those of the authors and do not necessarily represent those of their affiliated organizations, or those of the publisher, the editors and the reviewers. Any product that may be evaluated in this article, or claim that may be made by its manufacturer, is not guaranteed or endorsed by the publisher.

## References

[1] Adeoye O Broderick JP. Advances in the management of intracerebral hemorrhage. Nat Rev Neurol. (2010) 6:593–601. 10.1038/nrneurol.2010.146 20877400

[2] Members WG Lloyd-Jones D Adams RJ Brown TM Carnethon M Dai S. Heart disease and stroke statistics−2010 update: a report from the American Heart Association. Circulation. (2010) 121:e46–e215. 10.1161/CIRCULATIONAHA.109.192667 20019324

[3] Qureshi AI Mendelow AD Hanley DF. Intracerebral haemorrhage. Lancet. (2009) 373:1632–44. 10.1016/S0140-6736(09)60371-8 19427958 PMC3138486

[4] Gross BA Jankowitz BT Friedlander RM. Cerebral intraparenchymal hemorrhage: a review. Jama. (2019) 321:1295–303. 10.1001/jama.2019.2413 30938800

[5] Cordonnier C Demchuk A Ziai W Anderson CS. Intracerebral haemorrhage: current approaches to acute management. Lancet. (2018) 392:1257–68. 10.1016/S0140-6736(18)31878-6 30319113

[6] Ariesen M Claus S Rinkel G Algra A. Risk factors for intracerebral hemorrhage in the general population: a systematic review. Stroke. (2003) 34:2060–5. 10.1161/01.STR.0000080678.09344.8D 12843354

[7] Freytag E. Fatal hypertensive intracerebral haematomas: a survey of the pathological anatomy of 393 cases. J Neurol Neurosurg Psychiatry. (1968) 31:616. 10.1136/jnnp.31.6.616 5709848 PMC496432

[8] Kubo M Kiyohara Y Kato I Tanizaki Y Arima H Tanaka K. Trends in the incidence, mortality, and survival rate of cardiovascular disease in a Japanese community: the Hisayama study. Stroke. (2003) 34:2349–54. 10.1161/01.STR.0000090348.52943.A2 12958323

[9] Jiang B Wang W-z Chen H Hong Z Yang Q-d Wu S-p. Incidence and trends of stroke and its subtypes in China: results from three large cities. Stroke. (2006) 37:63–5. 10.1161/01.STR.0000194955.34820.78 16306469

[10] Flaherty ML Tao H Haverbusch M Sekar P Kleindorfer D Kissela B. Warfarin use leads to larger intracerebral hematomas. Neurology. (2008) 71:1084–9. 10.1212/01.wnl.0000326895.58992.27 18824672 PMC2668872

[11] Flibotte J Hagan N O'donnell J Greenberg S Rosand J. Warfarin, hematoma expansion, and outcome of intracerebral hemorrhage. Neurology. (2004) 63:1059–64. 10.1212/01.WNL.0000138428.40673.83 15452298

[12] Nicoll J McCarron M. APOE gene polymorphism as a risk factor for cerebral amyloid angiopathy-related hemorrhage. Amyloid. (2001) 8:51–5. 11676291

[13] Arima H Tzourio C Anderson C Woodward M Bousser M-G MacMahon S. Effects of perindopril-based lowering of blood pressure on intracerebral hemorrhage related to amyloid angiopathy: the PROGRESS trial. Stroke. (2010) 41:394–6. 10.1161/STROKEAHA.109.563932 20044530

[14] Mehndiratta P Manjila S Ostergard T Eisele S Cohen ML Sila C. Cerebral amyloid angiopathy–associated intracerebral hemorrhage: pathology and management. Neurosurg Focus. (2012) 32:E7. 10.3171/2012.1.FOCUS11370 22463117

[15] Mendelow AD Gregson BA Fernandes HM Murray GD Teasdale GM Hope DT. Early surgery versus initial conservative treatment in patients with spontaneous supratentorial intracerebral haematomas in the International Surgical Trial in Intracerebral Haemorrhage (STICH): a randomised trial. Lancet. (2005) 365:387–97. 10.1016/S0140-6736(05)70233-6 15680453

[16] Mendelow AD Gregson BA Rowan EN Murray GD Gholkar A Mitchell PM. Early surgery versus initial conservative treatment in patients with spontaneous supratentorial lobar intracerebral haematomas (STICH II): a randomised trial. Lancet. (2013) 382:397–408. 10.1016/S0140-6736(13)60986-1 23726393 PMC3906609

[17] Abdu E Hanley DF Newell DW. Minimally invasive treatment for intracerebral hemorrhage. Neurosurg Focus. (2012) 32:E3. 10.3171/2012.1.FOCUS11362 22463113

[18] Auer LM Deinsberger W Niederkorn K Gell G Kleinert R Schneider G. Endoscopic surgery versus medical treatment for spontaneous intracerebral hematoma: a randomized study. J Neurosurg. (1989) 70:530–5. 10.3171/jns.1989.70.4.0530 2926492

[19] Naff NJ Hanley DF Keyl PM Tuhrim S Kraut M Bederson J. Intraventricular thrombolysis speeds blood clot resolution: results of a pilot, prospective, randomized, double-blind, controlled trial. Neurosurgery. (2004) 54:577–84. 10.1227/01.NEU.0000108422.10842.60 15028130

[20] Teernstra OPM Evers S Lodder J Leffers P Franke C Blaauw G. Stereotactic treatment of intracerebral hematoma by means of a plasminogen activator: a multicenter randomized controlled trial (SICHPA). Stroke. (2003) 34:968–74. 10.1161/01.STR.0000063367.52044.40 12649510

[21] Zhang Z Li X Liu Y Shao Y Xu S Yang Y. Application of neuroendoscopy in the treatment of intraventricular hemorrhage. Cerebrovasc Dis. (2007) 24:91–6. 10.1159/000103122 17519550

[22] Kim YZ Kim KH. Even in patients with a small hemorrhagic volume, stereotactic-guided evacuation of spontaneous intracerebral hemorrhage improves functional outcome. J Korean Neurosurg Soc. (2009) 46:109. 10.3340/jkns.2009.46.2.109 19763212 PMC2744019

[23] Wang W-Z Jiang B Liu g-M Li D Lu C-Z Zhao Y-D. Minimally invasive craniopuncture therapy vs. conservative treatment for spontaneous intracerebral hemorrhage: results from a randomized clinical trial in China. Int J Stroke. (2009) 4:11–6. 10.1111/j.1747-4949.2009.00239.x 19236490

[24] Chen C-C Liu C-L Tung Y-N Lee H-C Chuang H-C Lin S-Z. Endoscopic surgery for intraventricular hemorrhage (IVH) caused by thalamic hemorrhage: comparisons of endoscopic surgery and external ventricular drainage (EVD) surgery. World Neurosurg. (2011) 75:264–8. 10.1016/j.wneu.2010.07.041 21492728

[25] Sun H Liu H Li D Liu L Yang J Wang W. An effective treatment for cerebral hemorrhage: minimally invasive craniopuncture combined with urokinase infusion therapy. Neurol Res. (2010) 32:371–7. 10.1179/016164110X12670144526147 20483003

[26] Naff N Williams MA Keyl PM Tuhrim S Bullock MR Mayer SA. Low-dose recombinant tissue-type plasminogen activator enhances clot resolution in brain hemorrhage: the intraventricular hemorrhage thrombolysis trial. Stroke. (2011) 42:3009–16. 10.1161/STROKEAHA.110.610949 21868730 PMC3356690

[27] Zhou H Zhang Y Liu L Han X Tao Y Tang Y. A prospective controlled study: minimally invasive stereotactic puncture therapy versus conventional craniotomy in the treatment of acute intracerebral hemorrhage. BMC Neurol. (2011) 11:1–8. 10.1186/1471-2377-11-76 21699716 PMC3142495

[28] Hanley DF Thompson RE Muschelli J Rosenblum M McBee N Lane K. Safety and efficacy of minimally invasive surgery plus alteplase in intracerebral haemorrhage evacuation (MISTIE): a randomised, controlled, open-label, phase 2 trial. Lancet Neurol. (2016) 15:1228–37. 10.1016/S1474-4422(16)30234-4 27751554 PMC5154627

[29] Vespa P Hanley D Betz J Hoffer A Engh J Carter R. ICES (intraoperative stereotactic computed tomography-guided endoscopic surgery) for brain hemorrhage: a multicenter randomized controlled trial. Stroke. (2016) 47:2749–55. 10.1161/STROKEAHA.116.013837 27758940 PMC6168060

[30] Hanley DF Lane K McBee N Ziai W Tuhrim S Lees KR. Thrombolytic removal of intraventricular haemorrhage in treatment of severe stroke: results of the randomised, multicentre, multiregion, placebo-controlled CLEAR III trial. Lancet. (2017) 389:603–11. 10.1016/S0140-6736(16)32410-2 28081952 PMC6108339

[31] Hanley DF Thompson RE Rosenblum M Yenokyan G Lane K McBee N. Efficacy and safety of minimally invasive surgery with thrombolysis in intracerebral haemorrhage evacuation (MISTIE III): a randomised, controlled, open-label, blinded endpoint phase 3 trial. Lancet. (2019) 393:1021–32. 10.1016/S0140-6736(19)30195-3 30739747 PMC6894906

[32] Barnes B Hanley DF Carhuapoma JR. Minimally invasive surgery for intracerebral haemorrhage. Curr Opin Crit Care. (2014) 20:148. 10.1097/MCC.0000000000000077 24553341 PMC5467438

[33] Backlund E-O von Holst H. Controlled subtotal evacuation of intracerebral haematomas by stereotactic technique. Surg Neurol. (1978) 9:99–101. 343283

[34] Kandel EI Peresedov VV. Stereotaxic evacuation of spontaneous intracerebral hematomas. J Neurosurg. (1985) 62:206–13. 10.3171/jns.1985.62.2.0206 3881565

[35] Hondo H Uno M Sasaki K Ebisudani D Shichijo F Tóth Z. Computed tomography controlled aspiration surgery for hypertensive intracerebral hemorrhage. Stereotact Funct Neurosurg. (1990) 54:432–7. 10.1159/000100248 2080361

[36] Matsumoto K Hondo H. CT-guided stereotaxic evacuation of hypertensive intracerebral hematomas. J Neurosurg. (1984) 61:440–8. 10.3171/jns.1984.61.3.0440 6379125

[37] Niizuma H Suzuki J. Stereotactic aspiration of putaminal hemorrhage using a double track aspiration technique. Neurosurgery. (1988) 22:432–6. 10.1227/00006123-198802000-00031 3281055

[38] Mould WA Carhuapoma JR Muschelli J Lane K Morgan TC McBee NA. Minimally invasive surgery plus recombinant tissue-type plasminogen activator for intracerebral hemorrhage evacuation decreases perihematomal edema. Stroke. (2013) 44:627–34. 10.1161/STROKEAHA.111.000411 23391763 PMC4124642

[39] Newell DW Shah MM Wilcox R Hansmann DR Melnychuk E Muschelli J. Minimally invasive evacuation of spontaneous intracerebral hemorrhage using sonothrombolysis. J Neurosurg. (2011) 115:592–601. 10.3171/2011.5.JNS10505 21663412 PMC3785332

[40] Sun S Liu X. A method for locating cerebral hematoma with head three-dimensional drawing. Chin J Stereot Funct Neurosurg. (2001) 14:183. 10.3969/j.issn.1008-2425.2001.03.116

[41] Luo J Sun S Quan W Cao Z Peng B Xie Q. A pilot study of therelationship between the drainage cannula and the cerebral angioarchitecture in patientsreceiving stereotactic cannula placement for hypertensive intracerebral hemorrhage. Chin J Neuromed. (2008) 7:1054–6. 10.3760/cma.j.issn.1671-8925.2008.10.021

[42] Luo J Sun S Cao Z Quan W Peng B Xiao G. CT-guided stereotactic evacuation for hypertensive intracerebral hemorrhage: analysis of 206 patients. Chin J Minim Invasive Neurosurg. (2009) 5:213–5.

[43] Ji Y Sun S Li G. The therapeutic effect of cubic oriented stereotactic hematoma aspiration on 32 cases with hypertensive cerebellar hemorrhage. Chin J Gen Pract. (2014) 12:1350–1.

[44] Sun Seditor. Cubic oriented stereotactic aspiration for hypertensive intracerebral hemorrhage. In: The 6th International Cerebrovascular Disease Summit Forum, Nanjing (2010).

[45] Fiorella D Arthur A Bain M Mocco J. Minimally invasive surgery for intracerebral and intraventricular hemorrhage: rationale, review of existing data and emerging technologies. Stroke. (2016) 47:1399–406. 10.1161/STROKEAHA.115.011415 27048700

[46] Ding D Przybylowski CJ Starke RM Street RS Tyree AE Crowley RW. A minimally invasive anterior skull base approach for evacuation of a basal ganglia hemorrhage. J Clin Neurosci. (2015) 22:1816–9. 10.1016/j.jocn.2015.03.052 26142050

[47] Przybylowski CJ Ding D Starke RM Crowley RW Liu KC. Endoport-assisted surgery for the management of spontaneous intracerebral hemorrhage. J Clin Neurosci. (2015) 22:1727–32. 10.1016/j.jocn.2015.05.015 26238692

[48] Labib MA Shah M Kassam AB Young R Zucker L Maioriello A. The safety and feasibility of image-guided brainpath-mediated transsulcul hematoma evacuation: a multicenter study. Neurosurgery. (2017) 80:515–24. 10.1227/NEU.0000000000001316 27322807

[49] Auer L Ascher P Heppner F Ladurner G Bone G Lechner H. Does acute endoscopic evacuation improve the outcome of patients with spontaneous intracerebral hemorrhage? Eur Neurol. (1985) 24:254–61. 10.1159/000115804 4040019

[50] Kuo L-T Chen C-M Li C-H Tsai J-C Chiu H-C Liu L-C. Early endoscope-assisted hematoma evacuation in patients with supratentorial intracerebral hemorrhage: case selection, surgical technique, and long-term results. Neurosurg Focus. (2011) 30:E9. 10.3171/2011.2.FOCUS10313 21456936

[51] Nagasaka T Tsugeno M Ikeda H Okamoto T Inao S Wakabayashi T. Early recovery and better evacuation rate in neuroendoscopic surgery for spontaneous intracerebral hemorrhage using a multifunctional cannula: preliminary study in comparison with craniotomy. J Stroke Cerebrovasc Dis. (2011) 20:208–13. 10.1016/j.jstrokecerebrovasdis.2009.11.021 20621516

[52] Spiotta AM Fiorella D Vargas J Khalessi A Hoit D Arthur A. Initial multicenter technical experience with the Apollo device for minimally invasive intracerebral hematoma evacuation. Operat Neurosurg. (2015) 11:243–51. 10.1227/NEU.0000000000000698 25714520

[53] Fiorella D Gutman F Woo H Arthur A Aranguren R Davis R. Minimally invasive evacuation of parenchymal and ventricular hemorrhage using the Apollo system with simultaneous neuronavigation, neuroendoscopy and active monitoring with cone beam CT. J Neurointerv Surg. (2015) 7:752–7. 10.1136/neurintsurg-2014-011358 25186443

[54] Tuhrim S Horowitz DR Sacher M Godbold JH. Volume of ventricular blood is an important determinant of outcome in supratentorial intracerebral hemorrhage. Crit Care Med. (1999) 27:617–21. 10.1097/00003246-199903000-00045 10199544

[55] Morgan TC Dawson J Spengler D Lees KR Aldrich C Mishra NK. The Modified Graeb Score: an enhanced tool for intraventricular hemorrhage measurement and prediction of functional outcome. Stroke. (2013) 44:635–41. 10.1161/STROKEAHA.112.670653 23370203 PMC6800016

[56] Gaberel T Magheru C Parienti J-J Huttner HB Vivien D Emery E. Intraventricular fibrinolysis versus external ventricular drainage alone in intraventricular hemorrhage: a meta-analysis. Stroke. (2011) 42:2776–81. 10.1161/STROKEAHA.111.615724 21817146

[57] Pang D Sclabassi RJ Horton JA. Lysis of intraventricular blood clot with urokinase in a canine model: part 3: effects of intraventricular urokinase on clot lysis and posthemorrhagic hydrocephalus. Neurosurgery. (1986) 19:553–72. 10.1227/00006123-198610000-00010 3491340

[58] Pang D Sclabassi RJ Horton JA. Lysis of intraventricular blood clot with Urokinase in a Canine Model: Part: canine intraventricular Blood Cast Model. Neurosurgery. (1986) 19:540–6. 10.1227/00006123-198610000-00008 3491338

[59] Pang D Sclabassi RJ Horton JA. Lysis of intraventricular blood clot with urokinase in a canine model: Part 2: *in vivo* safety study of intraventricular urokinase. Neurosurgery. (1986) 19:547–52. 10.1227/00006123-198610000-00009 3491339

[60] Huttner H Tognoni E Bardutzky J Hartmann M Köhrmann M Kanter IC. Influence of intraventricular fibrinolytic therapy with rt-PA on the long-term outcome of treated patients with spontaneous basal ganglia hemorrhage: a case–control study. Eur J Neurol. (2008) 15:342–9. 10.1111/j.1468-1331.2008.02077.x 18312407

[61] Vereecken KK Van Havenbergh T De Beuckelaar W Parizel PM Jorens PG. Treatment of intraventricular hemorrhage with intraventricular administration of recombinant tissue plasminogen activator: a clinical study of 18 cases. Clin Neurol Neurosurg. (2006) 108:451–5. 10.1016/j.clineuro.2005.07.006 16139422

[62] Kumar K Demeria DD Verma A. Recombinant tissue plasminogen activator in the treatment of intraventricular hemorrhage secondary to periventricular arteriovenous malformation before surgery: case report. Neurosurgery. (2003) 52:964–9. 10.1227/01.NEU.0000053028.06474.C6 12657195

[63] Nieuwkamp DJ Verweij BH Rinkel GJ. Massive intraventricular haemorrhage from aneurysmal rupture: patient proportions and eligibility for intraventricular fibrinolysis. J Neurol. (2010) 257:354–8. 10.1007/s00415-009-5323-z 19823896 PMC2837879

[64] Khan NR Tsivgoulis G Lee SL Jones GM Green CS Katsanos AH. Fibrinolysis for intraventricular hemorrhage: an updated meta-analysis and systematic review of the literature. Stroke. (2014) 45:2662–9. 10.1161/STROKEAHA.114.005990 25052321

[65] Longatti P Martinuzzi A Fiorindi A Maistrello L Carteri A. Neuroendoscopic management of intraventricular hemorrhage. Stroke. (2004) 35:e35–e8. 10.1161/01.STR.0000113736.73632.F6 14739413

[66] Basaldella L Marton E Fiorindi A Scarpa B Badreddine H Longatti P. External ventricular drainage alone versus endoscopic surgery for severe intraventricular hemorrhage: a comparative retrospective analysis on outcome and shunt dependency. Neurosurg Focus. (2012) 32:E4. 10.3171/2012.1.FOCUS11349 22463114

[67] Di Rienzo A Colasanti R Esposito D Della Costanza M Carrassi E Capece M. Endoscope-assisted microsurgical evacuation versus external ventricular drainage for the treatment of cast intraventricular hemorrhage: results of a comparative series. Neurosurg Rev. (2020) 43:695–708. 10.1007/s10143-019-01110-7 31069562

[68] Li Y Zhang H Wang X She L Yan Z Zhang N. Neuroendoscopic surgery versus external ventricular drainage alone or with intraventricular fibrinolysis for intraventricular hemorrhage secondary to spontaneous supratentorial hemorrhage: a systematic review and meta-analysis. PLoS ONE. (2013) 8:e80599. 10.1371/journal.pone.0080599 24232672 PMC3827437

[69] Zhu J Tang C Cong Z Yang J Cai X Liu Y. Endoscopic intraventricular hematoma evacuation surgery versus external ventricular drainage for the treatment of patients with moderate to severe intraventricular hemorrhage: a multicenter, randomized, controlled trial. Trials. (2020) 21:1–8. 10.1186/s13063-020-04560-3 32660530 PMC7359246

[70] Zhang J Cheng H Zhou S Huang L Li Y. 3D-printed model-guided endoscopic evacuation for basal ganglia hemorrhage. Sci Rep. (2020) 10:5196. 10.1038/s41598-020-62232-3 32251343 PMC7090061

[71] Wang Q Guo W Liu Y Shao W Li Z. Application of a 3D-printed navigation mold in puncture drainage for brainstem hemorrhage. J Surg Res. (2019) 245:99–106. 10.1016/j.jss.2019.07.026 31415935

[72] Kim SH Jang SH Lee CH Kang JH Byun WM Cho SH. Motor outcome according to diffusion tensor tractography findings in the early stage of intracerebral hemorrhage. Neurosci Lett. (2007) 421:142–6. 10.1016/j.neulet.2007.04.052 17566651

[73] Yoshioka H Horikoshi T Aoki S Hori M Ishigame K Uchida M. Diffusion tensor tractography predicts motor functional outcome in patients with spontaneous intracerebral hemorrhage. Neurosurgery. (2008) 62:97. 10.1227/01.NEU.0000311066.03121.B8 18300896

[74] Wu G Wang L Hong Z Mao Y Hu X. Effects of minimally invasive techniques for evacuation of hematoma in basal ganglia on cortical spinal tract from patients with spontaneous hemorrhage: observed by diffusion tensor imaging. Neurol Res. (2010) 32:1103–9. 10.1179/016164110X12656393665008 20483024

[75] Wang T Zhao Q Gu J Shi T Yuan X Wang J. Neurosurgery medical robot Remebot for the treatment of 17 patients with hypertensive intracerebral hemorrhage. Int J Med Robot Comp Assisted Surg. (2019) 15:e2024. 10.1002/rcs.2024 31267676

[76] Xiong R Li F Chen X. Robot-assisted neurosurgery versus conventional treatment for intracerebral hemorrhage: a systematic review and meta-analysis. J Clin Neurosci. (2020) 82:252–9. 10.1016/j.jocn.2020.10.045 33248949

